# Severity of Autism Symptoms and Degree of Attentional Difficulties Predicts Emotional and Behavioral Problems in Children with High-Functioning Autism; a Two-Year Follow-up Study

**DOI:** 10.3389/fpsyg.2017.02004

**Published:** 2017-11-14

**Authors:** Per N. Andersen, Kjell T. Hovik, Erik W. Skogli, Merete G. Øie

**Affiliations:** ^1^Department of Education and Social Work, Inland Norway University of Applied Sciences, Lillehammer, Norway; ^2^Division of Mental Health Care, Innlandet Hospital Trust, Sanderud, Norway; ^3^Division of Habilitation and Rehabilitation, Innlandet Hospital Trust, Lillehammer, Norway; ^4^Department of Psychology, University of Oslo, Oslo, Norway; ^5^Research Department, Innlandet Hospital Trust, Brumunddal, Norway

**Keywords:** High-Functioning Autism, Asperger's syndrome, autism spectrum disorder, emotional problems, behavioral problems, child behavior checklist, attention problems, verbal IQ

## Abstract

Children with autism often struggle with emotional and behavioral problems (EBP). This study investigated whether level of autism symptoms, attention problems or verbal IQ at baseline can predict EBP 2 years later in children with High-Functioning Autism (HFA). Thirty-four participants with HFA and 45 typically developing children (TD) (ages 9–16) were assessed with parent ratings of EBP, autism symptoms, attention problems, and a test of verbal IQ. The amount of autism symptoms and degree of attention problems at baseline significantly predicted EBP at follow-up, whereas verbal IQ did not. The findings from this study emphasize the importance of assessing and understanding the consequences of autism symptoms and attention problems when treating EBP in children with HFA. Furthermore, interventions aimed at improving ASD symptoms may positively affect the prevalence of EBP in children with HFA.

## Introduction

Children and adolescents with Autism Spectrum Disorders (ASD) often display emotional and behavioral problems (EBP) (Biederman et al., 2010; Gjevik et al., 2015; Hoffmann et al., 2016). More specifically, this group of children often report having so-called internalizing problems; i.e., sociability difficulties, anxiety, depression, thought problems, and withdrawal symptoms. Research has also indicated that children with ASD may exhibit externalizing problems, such as attention problems, hyperactivity, and conduct disorders (Gillberg and Billstedt, 2000; Simonoff et al., 2008; Mattila et al., 2010; Mayes et al., 2011; Hoffmann et al., 2016).

One of the most common comorbid conditions in children and adolescents with ASD is Attention/Deficit-Hyperactivity Disorder (ADHD) (Mattila et al., 2010; van Steensel et al., 2012; Stevens et al., 2016). There is no consensus, however, on whether ASD and ADHD should be considered different and distinct endophenotypes or share a similar underlying neurocognitive heterogeneity (Rommelse et al., 2010; van der Meer et al., 2014). There is also debate as to which extent ADHD rating scales aimed at neurotypical children are appropriate measures for assessing ADHD symptoms in children with ASD (Yerys et al., 2017). Core ASD symptoms such as the lack of social skills or the inability to disentangle oneself from special interests may inflate ratings of ADHD symptoms in children with ASD. Comorbid ASD and ADHD is associated with a higher degree of general psychopathology than ASD or ADHD alone (Holtmann et al., 2007; Jang et al., 2013). Externalizing behavior problems in particular have been found to be elevated in children and adolescents with comorbid ASD and ADHD (Guttmann-Steinmetz et al., 2009; Mulligan et al., 2009; Jang et al., 2013). Mansour et al. (2017) found that ADHD, but not ASD, was associated with more EBP as assessed by the Child Behavior Checklist (CBCL). Furthermore, children with ASD and concurrent ADHD symptoms had an increased prevalence of comorbid diagnoses, such as behavioral disorders, anxiety disorders and mood disorders. Other studies have reported that the level of autistic symptoms was related to higher EBP and more psychiatric comorbidity in a group of adolescents with autistic traits and in children diagnosed with ASD (Kanne et al., 2009; Rosenberg et al., 2011; Kotte et al., 2013).

Mayes et al. (2012) found that ADHD symptoms were common in children with ASD. One explanation for the common occurrence of comorbid ADHD symptoms in children with ASD is that these symptoms are secondary to difficulties with social functioning and/or that special interests lead to less sustained focus on other matters (Schatz et al., 2002). Another explanation is that attention problems in in children with ASD are a consequence of not being able to flexibly shift attentional focus to other matters. In contrast, children with ADHD commonly struggle to sustain focus on the same matter over time (Mayes et al., 2012). Interestingly, attention problems have been suggested as a common pathway to both ADHD and ASD (Visser et al., 2016; Sokolova et al., 2017). In any event, symptoms and disorders converge to some extent, and having similar features is more likely than not. An emphasis on symptom co-occurrence in children with developmental disorders is therefore important for clinical and research purposes (Leitner, 2014; Ronald et al., 2014).

Verbal IQ has been proposed to be an associating factor between ASD and ADHD symptom traits (Sokolova et al., 2017). The reason for this may be that verbal IQ is a prerequisite for engaging in social communication, which is often found impaired in people with ASD or ADHD (Geurts and Embrechts, 2008; Leonard et al., 2011). It may be an indicator of the ability to apply self-directed speech to help focus attention and exercise self-control. Better verbal IQ has been associated with less EBP in children with ASD (Mayes et al., 2011; Lord et al., 2015). There is also evidence that better verbal IQ mask difficulties with social and behavioral competence in children and adolescents with ASD, thus leading parents and others to report inflated social competence skills (Black et al., 2009). Large vocabularies and fluent speech may conceal more pragmatic language problems. The term “pragmatic language problems” refers to difficulties with coherence and mental state referencing, which is common in youth with ASD and can lead to social communication deficits (Rumpf et al., 2012). However, these subtler language difficulties are not necessarily detected in traditional tests of intelligence like the Wechsler tests (Simmons et al., 2014).

To our knowledge, no study has investigated whether the level of autism symptoms, inattention symptoms or verbal IQ in children and adolescents with ASD can predict EBP later on. A better understanding of the relationship of these problems and functions could support the development of assessment and intervention approaches to helping this vulnerable group of children and adolescents. Instability in comorbid symptom expression and concerns regarding inadequate explanatory power of internalizing and externalizing problems only, Caspi et al. (2014) argues that general symptoms of psychopathology, such as EBP, may be a better predictor of psychiatric disorders and adverse life events.

In the current study, children and adolescents with High-Functioning Autism (HFA-IQ above 70; Schopler, 1985; Gillberg, 1998; Wing, 1998) were recruited from Child and Adolescent Mental Health Centers (CAMHC), and follow-up assessments were conducted after 2 years. The levels of EBP, autism symptoms, inattention problems, and verbal IQ in this group of children are reported in previous publications (Andersen et al., 2015a,b, 2016). In this study, we investigated to what degree EBP at follow-up were related autism symptoms, inattention problems and verbal IQ at baseline. In addition, a hierarchical regression analysis was conducted to make stronger conclusions regarding any close relationships. The magnitude of core autism symptoms at baseline were expected to best predict degree of EBP at follow-up (Rosenberg et al., 2011; Yerys et al., 2017). We also expect higher attention symptoms to significantly predict elevated levels of EBP (Mansour et al., 2017). Based on previous research, we expect better verbal IQ to predict less EBP (Mayes et al., 2011; Lord et al., 2015).

## Methods

### Participants

Children and adolescents with HFA between 9 and 16 years were recruited from the Child and Adolescent Mental Health Centers (CAMHC) at Innlandet Hospital Trust (IHT) in Norway. The sample were included from all seven CAMHC in two Norwegian counties (Hedmark and Oppland) with a county-wide population of 375,000 people. See Andersen et al. (2015b) for details regarding recruitment procedure and demographic characteristics. At baseline (T1), all participants were assessed with separate face-to-face interviews of children and their parents (mainly the mother and on some occasions both parents) using the Schedule for Affective Disorders and Schizophrenia for School Age Children/Present and Lifetime version-2009 (K-SADS-PL; Kaufman et al., 1997). K-SADS-PL is a semi-structured diagnostic interview based on DSM-IV (American Psychiatric Association, 2000). It is made up of a screening interview and eight diagnostic supplements [affective disorders, psychotic disorders, anxiety disorders, behavioral disorders (ADHD), substance abuse disorders, eating disorders, tic disorders and ASD]. K-SADS-PL was supplemented with information from the Autism Spectrum Screening Questionnaire (ASSQ; Ehlers and Gillberg, 1993) and the ADHD rating scale IV (ARS-IV; DuPaul et al., 1998b). Diagnosis was confirmed if DSM-IV (American Psychiatric Association, 2000) criteria were met through an exhaustive evaluation of K-SADS-PL, parent reports, and self-reports together with information from teachers concerning academic and social functioning. This latter information is mandatory on referral to CAMHC.

Demographic and clinical characteristics are presented in Tables 1, 2.

**Table 1:** Demographic and clinical characteristics: means and standard deviations by group and assessment time.

	**Baseline (T1)**		**Follow-up (T2)**	
**Variable**	**HFA (*n* = 34)**	**TD (*n* = 45)**	**HFA (*n* = 34)**	**TD (*n* = 45)**
Sex (male/female)	28/6	29/16	28/6	29/16
Age	11.6 (2.0)	11.4 (1.5)	13.8 (2.0)	13.5 (1.4)
Time since T1 (mts)	–	–	25.6 (3.5)	24.9 (1.2)
Mother's education (yrs)	13.1 (2.6)	14.7 (2.4)	–	–
FSIQ^a^	99.9 (17.4)	104.5 (13.1)	98.5 (16.9)	106.5 (12.7)
CBCL total T-score^b^	63.8 (9.2)	38.2 (8.6)	55.9 (11.0)	36.3 (8.3)
ASSQ raw score^c^	20.6 (8.4)	1.7 (1.9)	20.4 (8.9)	1.2 (2.9)
ARS-IV inattention raw score^d^	12.7 (6.1)	1.5 (1.8)	9.9 (5.7)	1.6 (2.0)
VIQ^e^	98.6 (17.9)	99.4 (12.1)	97.5 (18.0)	102.6 (13.5)

*HFA, High-functioning Autism: TD, typically developing children*.

a *FSIQ, full scale IQ. IQ estimated measures from the Wechsler Abbreviated Scale of Intelligence (WASI)*.

b *CBCL, Child Behavior Checklist, total problems scale*.

c *ASSQ, Autism spectrum screening questionnaire*.

d *ARS-IV, ADHD Rating Scale version IV*.

e *VIQ, estimated verbal intelligence*.

**Table 2:** Demographic characteristics: univariate comparisons.

	**Baseline (T1)**		**Follow-up (T2)**	
**Variable**	**Group comparisons**		**Group comparisons**	
	**Chi-sq./F**	***p***	**Chi-sq./F**	***p***
Sex (male/female)	3.1	NS.	3.1	NS
Age	(1,77) 0.23	NS.	(1,77) 0.85	NS
Time since T1 (mts)	–	–	(1,77) 1.6	NS
Mother's education (yrs)	(1,77) 46.1	0.007	–	–
FSIQ^a^	(1,77) 1.8	NS.	(1,76) 5.6	0.020

*FSIQ, full scale IQ. IQ estimated measures from the Wechsler Abbreviated Scale of Intelligence (WASI)*.

a *Test scores from one participant with HFA were missing at T2*.

Twenty-eight in the HFA group were diagnosed with Asperger's syndrome (AS) and six with pervasive developmental disorder—not otherwise specified (PDD-NOS) at baseline. Five children with HFA also met criteria for ADHD. One child in the HFA group was using antipsychotics (aripiprazole = 5 mg) due to aggressive behavior and two children used psychostimulants (methylphenidate). Psychostimulants were discontinued 24 h prior to assessment.

The group of typically developing children (TD) (ages 9–16 years) were recruited from regular classes in local schools and was matched on age. The exclusion criteria for participation in both the HFA and TD groups were prematurity (<36 weeks), IQ estimate below 70, and any neurological disease. Further exclusion criteria for the TD group were no history of psychiatric disorder, dyslexia or head injury.

Sample size needed for running repeated measures/within-between group comparisons with an estimated effect size of 0.3 is *n* = 40 (G^*^Power 3.01.1; alpha = 0.05; power = 0.96).

After 2 years, a follow-up was conducted (T2). The re-assessment procedures at T2 were similar to T1. All HFA diagnoses were confirmed. One participant in the HFA group refused to participate at T2. One participant in the HFA group was using antipsychotic and antidepressant medication (quetiapine = 75 mg and sertraline 100 mg), another was using antidepressants (mianserin hydrochloride = 30 mg) and three were using psychostimulants (methylphenidate) at T2. Psychostimulants were discontinued 24 h prior to assessment.

Twenty-six children (76%) in the HFA group had received either help from specialized child/adolescent psychiatric outpatient clinics and/or extra help in school in the follow-up period. Data regarding interventions were missing for six children (17%). Psychoeducation for both patients, parents and the teachers are mandatory at the outpatients' clinics in order to facilitate socialization and reduce distress of the children. In addition, interdisciplinary treatment, special education groups, medication and supportive counseling are implemented if indicated.

All participants in the TD group were also reassessed at T2 (*n* = 45).

This study was carried out with approval from the Regional Committee for Medical Research Ethics in Eastern Norway (REK-Øst), and by the Privacy protection ombudsman for research at IHT. Both children (12 years and older) and parents gave their informed written consent prior to inclusion. Children below the age of 12 gave their verbal consent. Consents was given in accordance with the ethical principles given in the Declaration of Helsinki. All participants were of Caucasian origin.

### Measures used at T1 and T2

#### Measure of emotional and behavioral problems (EBP)

Parents completed the Achenbach's CBCL age 6–18 years, Norwegian translation, a parent-report measure on emotional and behavioral symptoms in children (Achenbach and Rescorla, 2001). The items measure specific EBP on a three point Likert scale. The first section of this inventory consists of seven competence items and the second section consists of 112 items on behavioral or emotional problems during the past 6 months. The 112 items on behavioral or emotional problems are made up of eight syndrome scales: anxious/depressed, withdrawn/depressed, somatic complaints, social problems, thought problems, attention problems, rule-breaking behavior, and aggressive behavior. These syndrome scales are classified into three broad band scales; Internalizing, Externalizing, and Total problems. Raw scores are converted to norm-referenced *T*-scores (*M* = 50, *SD* = 10). There are no national CBCL norms in Norway, but the scores from Norwegian children are lower compared to American children, and is a so-called low scoring country with a population mean well below T-score 50 (Kornør and Jozefiak, 2012). Hence, scores in the borderline area are graver than the American norm-referenced T-scores imply. Higher T-scores indicate higher degree of psychosocial problems. The CBCL has demonstrated good psychometric properties both internationally (Ivanova et al., 2007) and for the Norwegian version with good sensitivity (40–83%), specificity (70–94%), and internal consistency (Cronbach's alpha ≥ 0.8) (Nøvik, 1999; Kornør and Jozefiak, 2012). CBCL total score was of interest in this study

#### Measure of ASD symptoms

The ASSQ consists of 27 items rated on a three point Likert scale to be completed by either parent or teacher and was developed to screen for ASD. In the current study parent rating was used. The items encompass social interaction problems, communication problems and problems with restricted and repetitive behavior (Ehlers et al., 1999). ASSQ has excellent test-retest reliability, interrater reliability, sensitivity and specificity all ranging from 0.62 to 0.91 (Ehlers et al., 1999; Posserud et al., 2009). Higher scores indicate more problems.

#### Measure of inattention problems

The ADHD rating scale IV (ARS-IV; DuPaul et al., 1998b) was used to obtain parent rating of inattention symptoms. The ARS-IV consists of 18 questions regarding inattention (odd-numbered items) and hyperactivity/impulsivity (even-numbered items) in children and adolescents using a 4-point Likert scale. The psychometric properties of the ARS-IV have been reported to have good internal consistency (Cronbach's alpha ≥ 0.87) and intraclass correlation (≥0.87) (DuPaul et al., 1998a; Wyrwich et al., 2014). Higher scores indicate more problems.

#### Measure of general cognitive functioning (IQ)

To estimate full-scale IQ and verbal intelligence we used the Wechsler Abbreviated Scale of Intelligence-second edition (WASI; Wechsler, 2013).

### Data analyses

The data analyses were conducted using the statistical package SPSS for Windows version 24. Significant results are reported at *p* ≤ 0.05 and *p* ≤ 0.01 level. Demographic characteristics were investigated using the Chi-squared test for independence (sex) and one-way analysis of variance (ANOVA) (age, mother's education, time since T1 and IQ).

A Pearson bivariate correlation analysis was conducted to determine the association between ASD symptoms (T1), inattention problems (T1), verbal IQ (T1), and EBP (T2).

Finally, a hierarchical multiple regression analysis was performed to predict the proportion of variance in the dependent variable (EBP) at T2 that could be contributed to the independent variables (autism symptoms, inattention, and verbal IQ) at T1. Because of relatively few participants, and high symptom load in the HFA group on the dependent variable, we ran the analysis for the whole group. Blocks of predictors were entered into the model in four steps. The baseline model (model 1) had age at baseline as independent variable and served as a control variable for the subsequent analyses. In model 2 autism symptoms (ASSQ score) was added, model 3 added inattention symptoms (ARS-IV inattention score) and finally verbal intelligence (VIQ from WASI) was added in model 4. The increase in variance (Δ*R*^2^) was assessed for each block.

## Results

**Figure 1:**
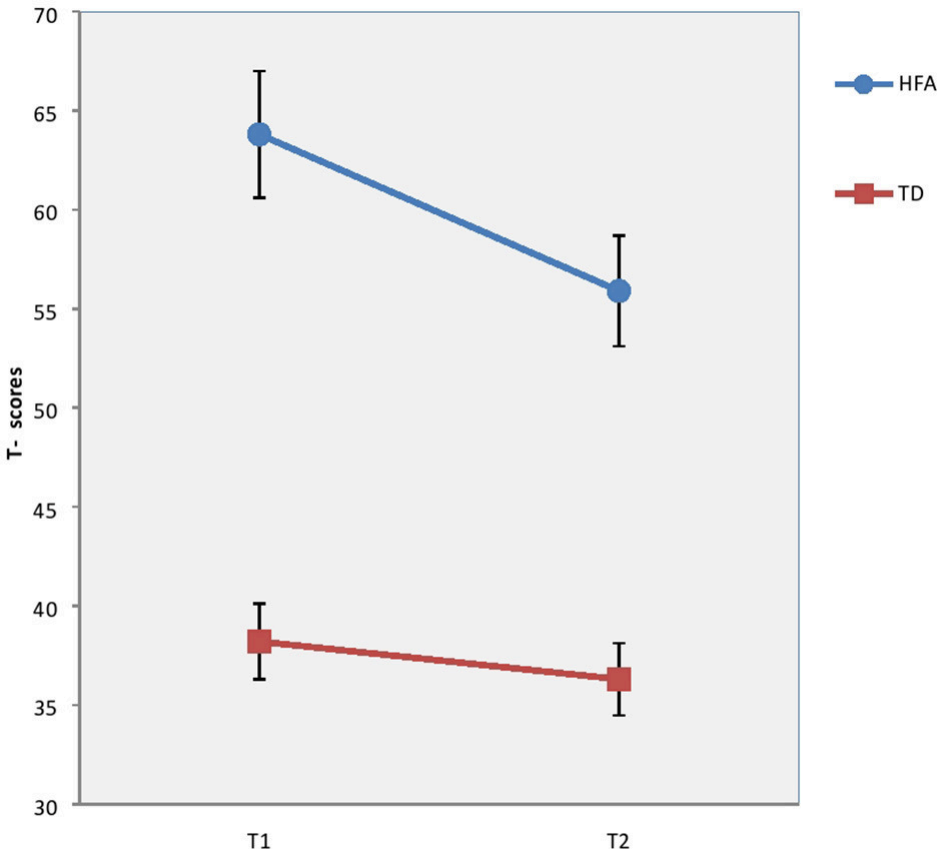
Results on Child Behaviour Checklist total problems scale at T1 and T2 in T-scores for children and with High-Functioning Autism (HFA) and typically developing children (TD). Vertical bar denotes 95% confidence intervals.

**Figure 2:**
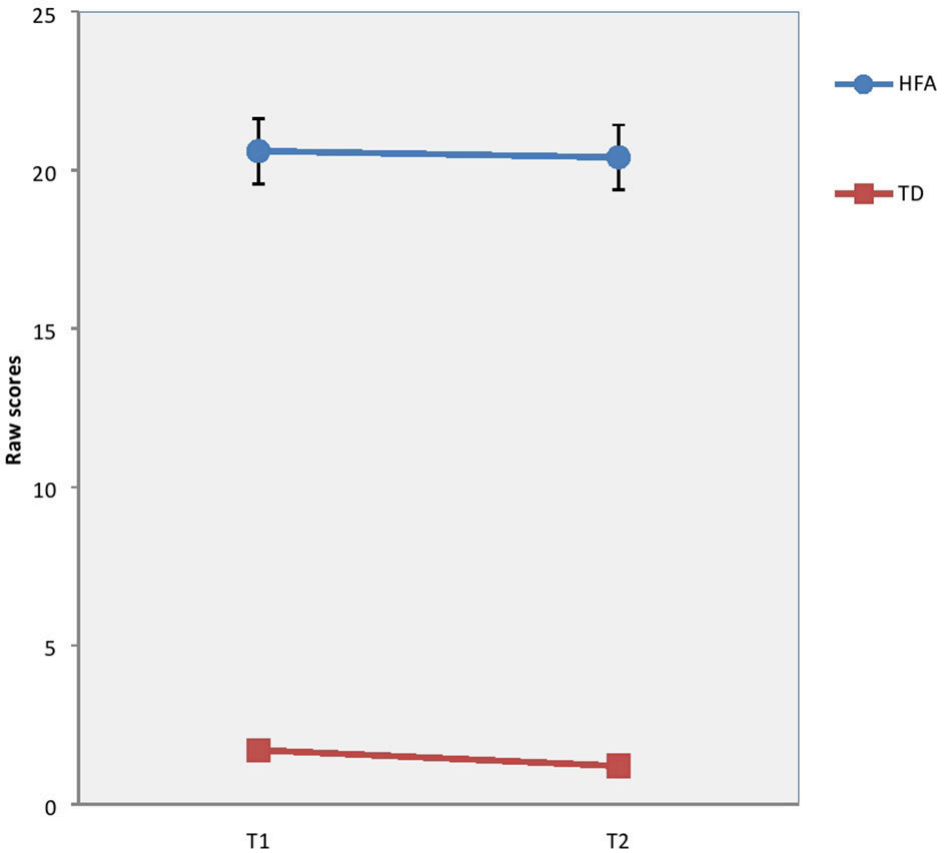
Results on Autism Spectrum Screening Questionnaire (ASSQ) at T1 and T2 in raw scores for children with High-Functioning Autism (HFA) and typically developing children (TD). Vertical bar denotes 95% confidence intervals.

**Figure 3:**
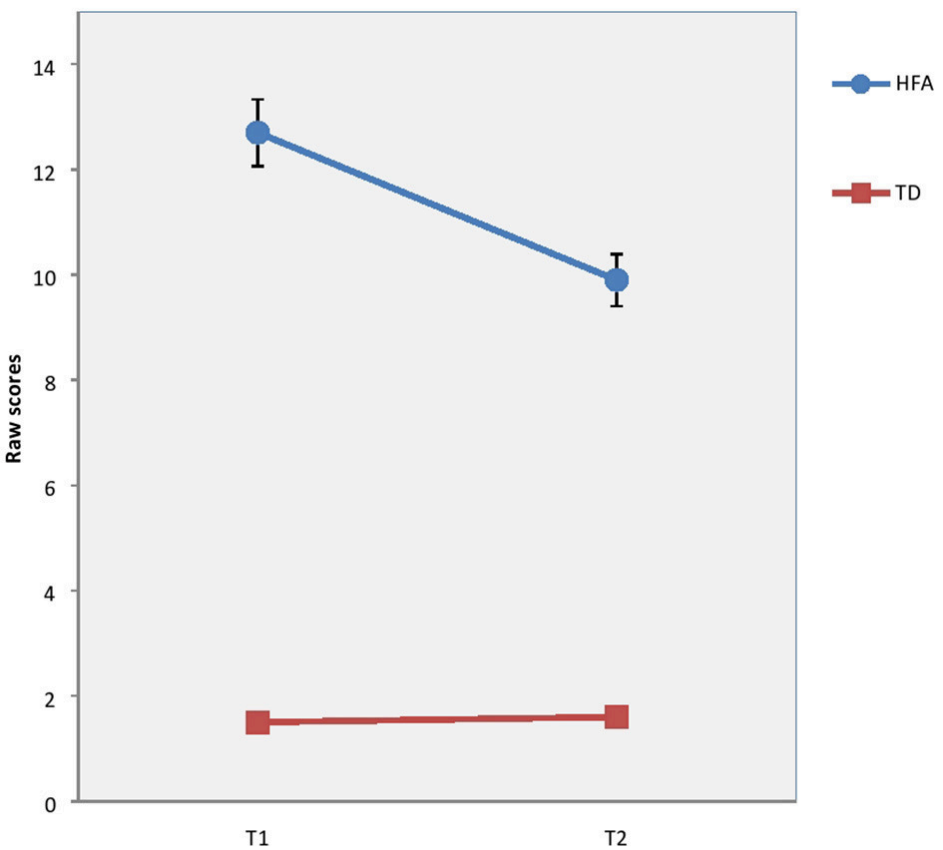
Results on ADHD Rating Scale IV (ARS-IV) at T1 and T2 in raw scores for children with High-Functioning Autism (HFA) and typically developing children (TD). Vertical bar denotes 95% confidence intervals.

There were no significant differences between groups concerning sex ratio (*p* = 0.079*)*, age (*p* = 0.637), or estimated full scale IQ (*p* = 0.183) at baseline. Although not significant the difference in ratio of males to females in the HFA group is close to those found in prevalence studies (Kadesjö et al., 1999; Isaksen et al., 2012; Surén et al., 2012). Mothers of TD children had significantly longer education than the clinical groups. However, the education level of the mothers of TD children was nearly equal to that of mothers of TD children in comparable studies in Norway (Heiervang et al., 2010). The longitudinal course of EBP, autism symptoms, degree of inattention and verbal IQ are previously reported (Andersen et al., 2015a, 2016), but are presented here as well (see Table 3, Figures 1–3), in order to help interpretation of the subsequent analyses.

**Table 3:** Results from Mixed Model ANOVA.

**Variable**	**Group**		**Time**		**Time × Group**		
	**F**	***p***	**F**	***p***	**F**	***p***	$\eta_{\text{p}}^{2}$
CBCL total^a^	(1,74) 142.1	<0.001	24.1	<0.001	9.7	0.003	0.115
ASSQ^b^	(1,76) 312.0	<0.001	0.193	NS	0.054	NS	0.001
ARS-IV inattention^c^	(1,77) 145.3	<0.001	7.6	0.007	9.6	0.003	0.111
VIQ^d^	(1,74) 0.7	NS	1.3	NS	6.2	0.015	0.077

a *CBCL, Child Behavior Checklist; total problems scale, two missing from children in the HFA group and one from the TD*.

b *ASSQ, Autism spectrum screening questionnaire, one missing from the TD*.

c *ADHD rating scale version IV*.

d *VIQ, estimated verbal intelligence, three missing from children in the HFA group*.

The correlation analysis in the current study (see Table 4) revealed a strong correlation between EBP (CBCL total problems) at T2 and ASD symptoms (ASSQ score) at T1 (*r* = 0.74*, p* < 0.001) and inattention problems (ARS-IV) (*r* = 0.71*, p* < 0.001). There was also a strong correlation between ASD symptoms (ASSQ score) and inattention problems (ARS-IV score) at T1 (*r* = 0.84*, p* < 0.001).

**Table 4:** Correlations.

	**T2 CBCL total problems**	**T1 ASSQ**	**T1 ARS-IV Inattention**	**T1 VIQ**
T2 CBCL^a^ total problems	1	0.741^**^	0.710^**^	−0.143
T1 ASSQ^b^	0.741^**^	1	0.842^**^	−0.108
T1 ARS-IV inattention^c^	0.710^**^	0.842^**^	1	−0.187
T1 VIQ^d^	−0.143	−0.108	−0.187	1

** *Correlation is significant at the <0.01 level (2-tailed)*.

a *CBCL, Child Behavior Checklist, total problems scale*.

b *ASSQ, Autism spectrum screening questionnaire*.

c *ARS-IV ADHD rating scale-inattention subscale*.

d *Verbal IQ estimated measure from Wechsler Abbreviated Scale of Intelligence*.

**Figure 4:**
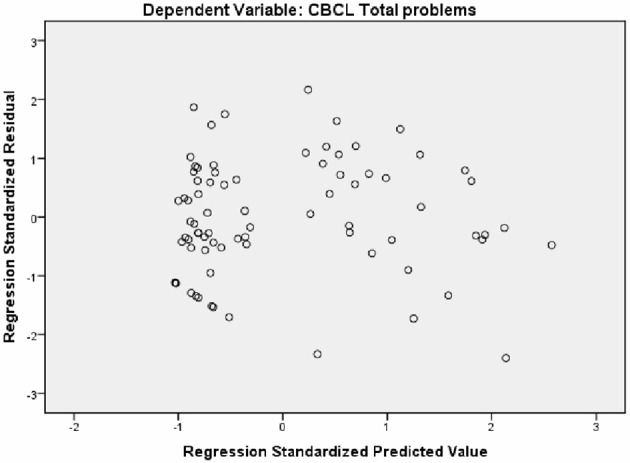
Scatterplot of standardized predicted values against standardized residuals for the regression model.

A hierarchical multiple regression analysis was performed to predict the proportion of variance in the dependent variable (EBT) that could be attributed to the independent variables (autism symptoms, inattention and verbal IQ). Results from the hierarchical regression are presented in Table 5 and Figure 4. Adding ASD symptoms (ASSQ) into the model in addition to the baseline (age) model gave a significant improvement in model fit (Δ*R*^2^ = 0.54, Δ*F* = 89.74, *p* < 0.001). This was also the case when inattention (ARS-IV) was entered into the model (Δ*R*^2^ = 0.024, Δ*F* = 4.14, *p* = 0.045). Finally, inclusion of verbal IQ did not significantly improve the model (Δ*R*^2^ = 0.001, Δ*F* = 0.22, *p* = 0.644).

**Table 5:** Summary of hierarchical regression analysis for CBCL total problems (*n* = 78).

**Variable**	**Model 1**	**Model 2**	**Model 3**	**Model 4**
Constant	34.851	38.822	36.752	40.173
Age	0.86	−0.27	−0.134	−0.138
ASSQ		0.919^**^	0.615^*^	0.623^*^
ARS-IV inattention			0.562^*^	0.539
WASI-VIQ				−0.034
*R*^2^	0.012	0.550	0.574	0.575
*F*	0.926	45.87^**^	33.25^**^	24.72^**^
Δ*R*^2^		0.54	0.024	0.001
Δ*F*		89.74^**^	4.14^*^	0.22

The table displays the unstandardized regression coefficients for the five models.

* p < 0.05.

** *p < 0.01. ASSQ, Autism Spectrum Screening Questionnaire: ARS-IV attention; ADHD Rating Scale–IV Inattention subscale (odd-numbered items): WASI-VIQ, Wechsler Abbreviated Scale of Intelligence-estimated Verbal IQ*.

## Discussion

As expected, the level of autism symptoms at baseline were the best predictor of EBP in our group of children with HFA (EBP; CBCL total problems) at follow-up. Also as expected, attention problems had an independent impact on EBP, although considerably less compared to the level of autism symptoms. Including verbal IQ in the model did not add to our understanding of the factors contributing to EBP in children and adolescents with ASD.

### Autism symptoms

The results emphasize the close relationship between ASD symptoms and EBP. The predictive validity of ASD symptoms at baseline on EBP at follow-up, was large with a 54% increase in model fit. This result emphasizes the importance of severity of autism symptoms as a main contributor to EBP in these children. Emphasis on comorbid symptoms must not overshadow the importance of severity of the main characteristics of ASD. Despite treatment in child and adolescents' outpatient clinics during the follow-up period, ASD symptoms did not decrease in the children with HFA. Even though the level of EBP had decreased significantly, the level was still elevated compared to TD. In addition, considering the low population mean on the CBCL in Norway (Kornør and Jozefiak, 2012), these symptoms may be more impairing than the T-scores indicate on face value. These results corroborate previous studies reporting an association between ASD symptoms and EBP as measured by the Behavioral Assessment System for Children-second edition (BASC-2) and the CBCL (Kanne et al., 2009; Kotte et al., 2013). Our data may provide evidence of some overlap between questionnaires based on associations between the ASSQ and the CBCL parent rating scale, as well as the ARS-IV and the CBCL parent rating scale. As illustrated in Table 3, CBCL total problems scale correlated strongly with ASSQ (*r* = 0.74) and ARS-IV (*r* = 0.71). This overlap between ASSQ, ARS-IV, and CBCL may thus result in some circularity when interpreting our findings.

### Attention problems

The relatively low contribution (2.4%) of attention problems (as reported by parents) to model fit may indicate that attention difficulties in HFA are not merely a part of a comorbid disorder, such as ADHD, but a core symptom of autism. The level of attention problems for children with HFA was higher than for TD children and significantly correlated with EBP, despite a relatively small impact on the regression model. As such, parent reported attention problems in their children with ASD may represent a part of the shared attentional problems in ASD and ADHD (Kotte et al., 2013; Sokolova et al., 2017). It is also possible that the ARS-IV is not adequately designed to disentangle the inattention problems seen in ASD, and that the inattention problems captured in ARS-IV actually reflect the variability in ASD symptoms (Yerys et al., 2017). As ADHD manifests itself in settings requiring social and communication skills, ADHD together with ASD enhance vulnerability for EBP (Mansour et al., 2017). However, as respondents interpret questionnaires into the context in which they are given, answers may be given to questions accordingly. For instance, questions in an ADHD assessment tool may be interpreted as symptoms of ADHD while they may simultaneously represent ASD problems (e.g., does not listen, has problems sustaining attention) (Yerys et al., 2017). Furthermore, as ADHD largely manifests itself in settings requiring social and communication skills, it may not be easy to disentangle ADHD symptoms from ASD symptoms. Moreover, there is a possibility to “overemphasize” comorbid symptoms that are possibly a result of core ASD symptoms. An indication of this symptom overlap is the rather large correlation between ASSQ and ARS-IV Inattention.

Inattention problems in ASD may also be related to Executive Functions (EF) as these have been found to be impaired in both children with ASD and ADHD (Hosenbocus and Chahal, 2012). EF refers to the neurocognitive skills and subprocesses underlying purposeful and goal-directed behavior (Zelazo and Müller, 2011). Especially the EF subprocess inhibition which is directly related to attentional capacity have been linked to comorbid ADHD symptoms in ASD (Wallace et al., 2016).

### Verbal intelligence

As previously referred, verbal abilities and verbal intelligence, has been reported as associated with EBP in ASD (Mayes et al., 2011; Lord et al., 2015). However, contrary to this, verbal IQ did not contribute significantly to EBP in our model. Most of the children with HFA in this study were diagnosed with Aspergers' syndrome, which excludes a significant delay in language acquisition (American Psychiatric Association, 2000). As such, the characteristics of our sample may not sufficiently reflect the suggested association between verbal IQ and EBP. However, achieving the age appropriate level of language acquisition or having verbal IQ within the normal range, may conceal more subtle language problems such as pragmatic difficulties. Traditional measures of verbal IQ do not include important pragmatics such as topic initiation, topic maintenance, turn taking, non-verbal signals, amount of talk and intensity, which are embedded in social communication (Simmons et al., 2014). Contrary to this, Whyte and Nelson (2015) found that verbal IQ were associated with pragmatic language and that better verbal IQ was a protective factor against social communication deficits. The participants in their study was younger than in the present study and they used another measure of verbal IQ (KBIT2) which may be a reason for different results.

## Conclusion

In conclusion, the results show the level of autistic symptoms as the main predictor of EBP in children and adolescents with HFA after 2 years. Although highly correlated, attention problems did not seem to be an important contributor of EBP. The latter finding stresses the importance of applying assessment tools with adequate psychometric properties for ASD when assessing inattention.

### Clinical implications

Interventions aimed at improving ASD symptoms, such as for example social skills training and peer-mediated interventions, may have positive outcomes for the prevalence of EBP seen in children and adolescents with HFA. Attention problems are elevated in the HFA group and should be regarded as clinically relevant. When assessing attention problems, tools intended for ADHD screening should be used carefully and combined with other measures such as neuropsychological tests and detailed anamnestic information.

### Strengths and limitations of the study

The longitudinal design and low dropout rate are strengths in the current study. The large age span (9–16 years at T1) might represent a limitation, but small standard deviations in age reflect the fact that most participants were pre-adolescents at T1. Another potential limitation is the relatively small sample size and that it was drawn from a clinical population, and represents those who are willing to seek help and might have higher rates of attention than the general ASD population. This study focused on symptoms of autism and attention, and we cannot generalize these findings to those with ASD and clinical diagnoses of ADHD. Results are also restricted to those with an IQ above 70. Inferences regarding sex differences was not possible due to few girls in the ASD group. It is also a limitation that we made use of other diagnostic instruments than ADI/ADOS, which are commonly used in research. Furthermore, we did not control for interventions in the follow-up period.

## Author contributions

PA: collected the data material, did the analyses with interpretation, and was in charge of drafting the manuscript. ES, KH, and MØ: collected the data material, contributed to data analyses and interpretation, and wrote parts of the manuscript. All authors proofread and revised the manuscript and gave approval to publication.

### Conflict of interest statement

The authors declare that the research was conducted in the absence of any commercial or financial relationships that could be construed as a potential conflict of interest.
